# Beneficial Effects of Caloric Restriction on Chronic Kidney Disease in Rodent Models: A Meta-Analysis and Systematic Review

**DOI:** 10.1371/journal.pone.0144442

**Published:** 2015-12-22

**Authors:** Xiao-meng Xu, Guang-yan Cai, Ru Bu, Wen-juan Wang, Xue-yuan Bai, Xue-feng Sun, Xiang-mei Chen

**Affiliations:** 1 Department of Nephrology, Chinese PLA General Hospital, Chinese PLA Institute of Nephrology, State Key Laboratory of Kidney Diseases, National Clinical Research Center for Kidney Diseases, Beijing, China; 2 School of Medicine, Nankai University, Tianjin, China; University of Utah School of Medicine, UNITED STATES

## Abstract

**Background:**

Numerous studies have demonstrated the life-extending effect of caloric restriction. It is generally accepted that caloric restriction has health benefits, such as prolonging lifespan and delaying the onset and progression of CKD in various species, especially in rodent models. Although many studies have tested the efficacy of caloric restriction, no complete quantitative analysis of the potential beneficial effects of reducing caloric intake on the development and progression of CKD has been published.

**Methods:**

All studies regarding the relationship between caloric restriction and chronic kidney diseases were searched in electronic databases, including PubMed/MEDLINE, EMBASE, Science Citation Index (SCI), OVID evidence-based medicine, Chinese Bio-medical Literature and Chinese science and technology periodicals (CNKI, VIP, and Wan Fang). The pooled odds ratios (OR) and 95% confidence intervals (95% CI) were calculated by using fixed- or random-effects models.

**Results:**

The data from 27 of all the studies mentioned above was used in the Meta analysis. Through the meta-analysis, we found that the parameter of blood urea nitrogen, serum creatinine and urinary protein levels of the AL group was significant higher than that of the CR group, which are 4.11 mg/dl, 0.08mg/dl and 33.20mg/kg/24h, respectively. The incidence of the nephropathy in the caloric restriction (CR) group was significantly lower than that in the ad libitum—fed (AL) group. We further introduced the subgroup analysis and found that the effect of caloric restriction on the occurrence of kidney disease was only significant with prolonged intervention; the beneficial effects of CR on the 60%-caloric-restriction group were greater than on the less-than-60%-caloric-restriction group, and caloric restriction did not show obvious protective effects in genetically modified strains. Moreover, survival rate of the caloric restriction group is much higher than that of the ad libitum—fed (AL) group.

**Conclusions:**

Our findings demonstrate for the first time that compared with the AL group, the caloric restriction indeed decreased urea nitrogen, creatinine, urine protein, incidence of kidney diseases and increased the survival rate on 700~800 days.

## Introduction

The incidence of CKD has gradually increased which resulted in 956,000 deaths in 2013 up from 409,000 deaths in 1990 and the cost of health services for patients with CKD is also a huge financial burden to our government and society which is about 1.8 times higher than for patients without CKD [1]. Aging-related nephropathy plays a critical role in CKD. Compared with younger age groups, elderly patients have a greater risk of developing CKD. In 1998, 47% of dialysis patients were over 65 years of age, and by 2000, this percentage rose to over 60%. Indeed, geriatric patients are at an increased risk of renal dysfunction from many causes that are inherent to aging, such as decreases in physiological function and anatomical and physiological alterations. The number of glomeruli in an individual ranges from 250,000 to more than 1.5 million per kidney at birth, and this number decreases with age at a rate of approximately 6,752 glomeruli/year after 18 years of age [2]. In addition, renal mass decreases from >400 g during the 3rd and 4th decades to <300 g by the 9th decade [3]. In addition to aging-related diseases, diabetic kidney disease is another important component of CKD. Diabetic nephropathy is the leading cause of CKD in patients beginning renal replacement therapy, and it affects approximately 40% of both type 1 and type 2 diabetic patients.

Based on the above observations, it is clear that CKD has become a major cause of human mortality. If we cannot prevent or mitigate CKD, it will reduce human health and welfare and cause huge financial burdens to society and family. The first line of treatment is to adopt a healthy lifestyle. However, what kind of lifestyle will yield the biggest benefit? How great a benefit can people expect from such a lifestyle change? The large individual variability in response to diet and exercise represents a huge challenge in clinical practice.Since the first paper published by McCay [4] in 1935 demonstrated the beneficial effect of caloric restriction, numerous studies have demonstrated the life-extending effect in laboratory rats [5–7]. It is generally accepted that caloric restriction has health benefits, such as prolonging lifespan and delaying the onset and progression of cardiovascular disease, Alzheimer's disease, chronic metabolic disorders and various other chronic diseases in rodent models [8–10]. However, only in the last two decades have researchers reported a reduction in the age-associated decline in renal function, decreases in the incidence of renal lesions, and a reduction in the age-associated changes in renal morphology possibly by restricting the food intake of laboratory rat strains, including Wistar, Sprague-Dawley and Fischer 344 [11–13]

Although many studies have tested the efficacy of caloric restriction, However, data available on the effect of dietary restriction on CKD are conflicting. Divergent results have been reported [13–14] and no complete quantitative analysis of the potential beneficial effects of reducing caloric intake on the development and progression of CKD has been published. In this analysis, we reviewed, for the first time, the existing literature describing CKD and analyzed the beneficial effects of caloric restriction by evaluating biochemical indexes and the occurrence of CKD. Therefore, we believe that this study will explore the efficiency of CR on CKD and be the good guide of the new treatments for kidney disease in the near future.

## Materials and Methods

### 2.1 Search strategy

The study was conducted according to the Preferred Reporting Items for Systematic Reviews and Meta-Analyses criteria (PRISMA) as shown in S1 File. We searched the following databases: PubMed/MEDLINE, EMBASE, Science Citation Index (SCI), Evidence-based Medicine Reviews (OVID), Chinese Biomedical Literature and Chinese Science and Technology Periodicals (CNKI, VIP, and Wan Fang) (May 2014). The references of recent review articles were also surveyed for additional studies. Two researchers independently conducted searches to ascertain the conformity of the inclusion criteria. If the decision to include or exclude an article could not be made by reading the title and the abstract alone, the full text of the article was carefully reviewed.

The following medical subject heading items and free-text words were employed: renal insufficiency, renal failure, renal injury, kidney function, kidney injury, nephropathy, disease onset, incidence of the disease AND caloric restriction, diet restriction, and nutrition.

### 2.2 Inclusion and exclusion criteria

The inclusion/exclusion criteria were as follows. (i) The experiment contained both a control group and a caloric-restricted group. (ii) Protein restriction and some specific nutrient restrictions were excluded. (iii) Kidney functional parameters (i.e., serum urea nitrogen, serum creatinine, and urinary protein excretion) and the incidence of kidney disease was reported and could be extracted from tables or figures. (iv) The study included no other confounding treatments, e.g., drugs or exercise. (v) The same data set was only used once in the meta-analysis, even if it appeared in multiple publications, to avoid misleading results. (vi) If more than two matched experimental groups were included, all of the groups in the study were included. (vii) When multiple experimental groups with the same degree of CR were available (e.g., different nutritional components) within the same study, we selected the experimental group with the experimental protocol that was most comparable to that of the control group.

### 2.3 Assessment of methodological quality

The quality of the methods of the included studies was assessed based on the ARRIVE guidelines (15) (16). Methodological quality was assessed based on sample size, whether random allocation into treatment and control groups was used, husbandry conditions (e.g., breeding programs, light/dark cycle, temperature, type of food, access to food, access to water, and environmental enrichment), whether the investigators were blind to the treatment compliance with animal welfare regulations, statement of potential conflicts of interests, and whether the study appeared in a peer-reviewed publication. Each study was assessed by 2 independent researchers and scored on a scale from 0 (lowest) to 7 (highest) points.

### 2.4 Data collection

Using a custom data collection form, the following data were extracted: rat strain, rat sex, number of rats in the group treated with CR, number of rats in the ad libitum—fed group (AL), percentage of caloric restriction, intervention type, timing of intervention, duration of intervention, and outcomes. Additionally, the mean outcome, SD, and number of rats in each group were extracted from the data. In experiments where different groups were subjected to different percentages of caloric restriction, we considered the groups to be independent. When outcomes were measured at several time points, groups were considered to be different only if the CR group was different. However, if they were compared with the same group, only the final measurement was included.

### 2.5 Statistical analysis

All statistical analyses were conducted using Review Manager Software recommended by the Cochrane Collaboration (Version 5.2. Copenhagen: The Nordic Cochrane Centre, the Cochrane Collaboration, 2012). The results were expressed as odds ratios (ORs) for dichotomous data and mean differences (MD) for continuous data, with 95% confidence intervals (CIs). Heterogeneity among included trials was analyzed using the chi-squared (χ^2^, or Chi^2^) test. If a P-value >0.10 was found, indicating no heterogeneity among the included trials, the Mantel-Haenszel (M-H) method using a random model was conducted to perform a meta-analysis of the dichotomous data, and the inverse variance (IV) method with a random model was used for continuous data. The data were aggregated using a random effects model because of the high heterogeneity of the systematic review of the animal experiments.

## Results

### 3.1 Selection of included studies

**Fig 1:**
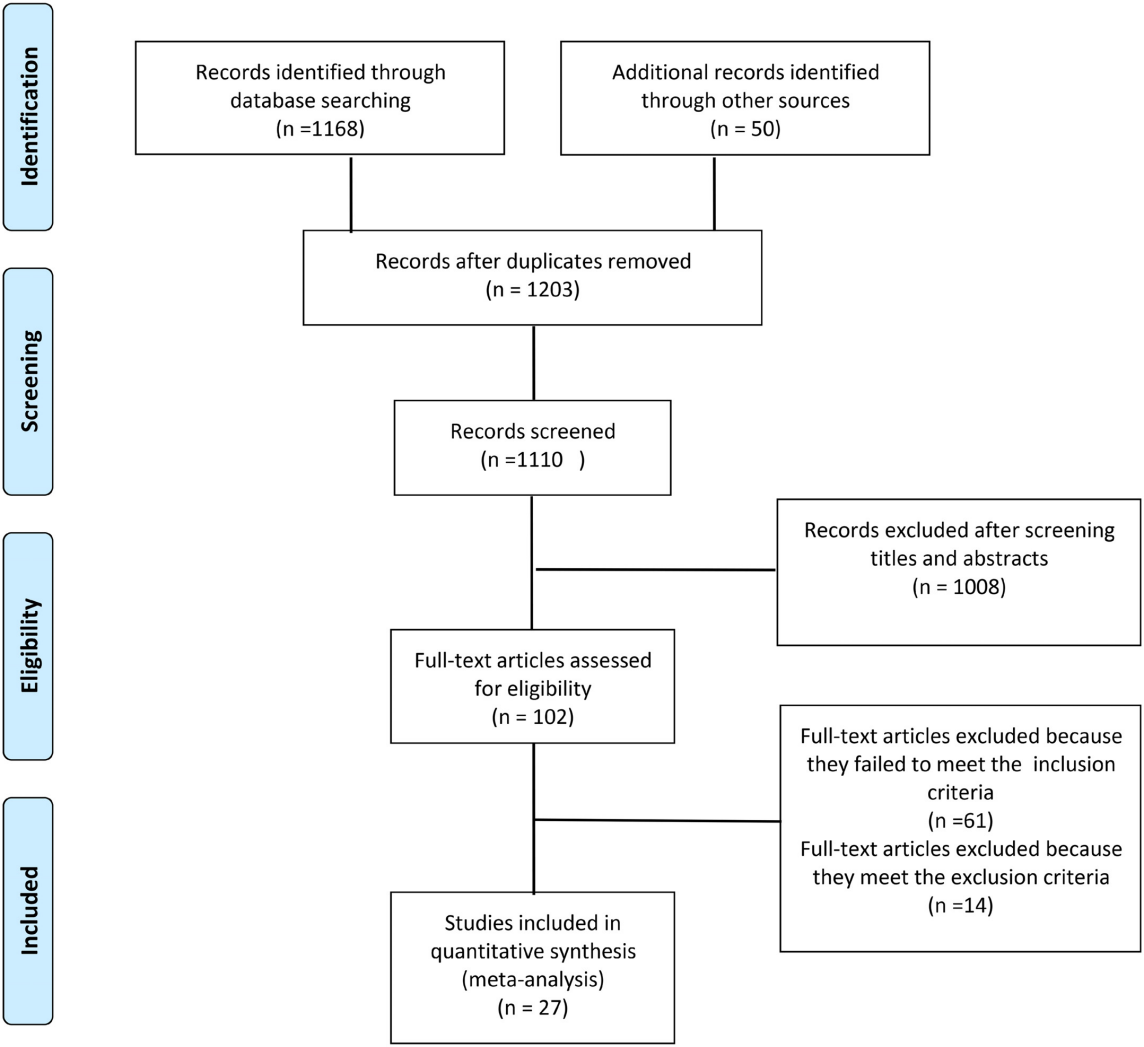
Summary of the process for identifying candidate studies.

The search identified 1168 articles, 1056 of which were excluded after screening the titles and abstracts. After screening, 102 relevant citations remained for further review. We excluded 61 of these 102 studies because they either did not include the necessary data or did not meet the inclusion criteria. Finally, 27 citations (S2 File) were used in the meta-analysis (Fig 1).

### 3.2 The methodological quality of the included studies

A summary of the methodological domain assessment for each study is shown in Table 1.

**Table 1:** Risk-of-bias summary. A review of the author’s judgments about each risk-of-bias item for each included study. Points ranged from 0 to 7.

	Sample size calculation	Random allocation to treatment or control	Husbandryconditions	Blinding of outcome assessment	Compliance with animal welfare regulations	Statement of potential conflict of interests	Peer-reviewed publication	Score
**Arthur V.Everitt 1982**		√	√				√	3
**Byung Pal Yu 1982**		√	√	√			√	4
**Chen J 2008**		√	√		√		√	4
**Cui J 2013**		√	√	√	√		√	5
**David C Tapp 1989**		√	√				√	3
**Duffy PH 2008**		√	√		√		√	4
**Dutra MF 2012**		√	√		√		√	4
**J Hempe 2012**		√	√		√		√	4
**Keenan KP 2000**		√	√	√	√		√	5
**Kobayashi S 1992**		√	√		√		√	4
**Kume S 2010**		√	√			√	√	4
**Lezcano EJ 2014**		√	√	√	√		√	5
**Mark DA 1984**		√	√				√	3
**Masoro EJ 1989**		√	√		√		√	4
**Nakano D 2011**		√	√			√	√	4
**Nangaku M 2005**		√	√	√	√	√	√	6
**Yichun Ning 2013**		√	√		√		√	4
**Patricia R Johnson 1997**		√	√		√		√	4
**Podkowka-Sieczka 2009**		√	√	√	√		√	5
**Sell DR 2000**		√	√		√		√	3
**Shimokawa I 2003**		√	√				√	3
**TERESA A. DAVIS 1983**		√	√				√	3
**Tikoo K 2007**		√	√		√		√	4
**Wiggins JE 2005**		√	√		√		√	4
**Zha Y 2008**		√	√				√	3
**Sarah M. Tucker 1976**		√			√		√	3
**Jennifer R. Wyndham 1983**		√	√				√	3

### 3.3 Study characteristics

The characteristics of the selected studies are shown in Table 2. The experimental animal species were predominantly F344 rats, SD rats or Wistar rats, but some other genetically modified rodents were also used. In the selected studies, the caloric intake of the calorie-restricted group was between 50~72% of the caloric intake of ad libitum-fed the group. Other caloric restriction methods (fasting and pair-fed) were also included in our meta-analysis. The duration of the interventions ranged from 1 week to 2 years or more, and young, middle-aged and old were used as the intervention times in these studies. Additionally, biological parameters (incidence of chronic nephropathy, urinary protein, BUN, Scr, survival rate) served as study endpoints.

**Table 2:** The design characteristics of the include studies.

Study	Rat type	Sex	N(CR)	N(AL)	Percentage of caloric restriction	Duration of intervention	Timing of intervention	Outcomes (relevant to the analysis)
**Arthur V.Everitt 1982**	wistar rats	male	8	8	50%	250day	28 days of age	Urinary protein,survival rate
**Byung Pal Yu 1982**	F344 rats	male	531	531	60%	6 months,12 months, 18 months,24 months	28 days of age	survival rate
**Chen J 2008**	F344 rats	male	11	11	60%	6 month	12 month of age	Urine protein
**Cui J 2013**	F344 rats	male	16	16	60%	21 month	3 month	Scr
**David C Tapp 1989**	F344 rats	male	9	8	63.60%	1 week	After surgery	Urinary protein
**Duffy PH 2008**	SD rats	male	20	20	69%	52 week	6 weeks of age	Incidence of chronic nephropathy,survival rate
	SD rats	male	40	60	69%	108 week	6 weeks of age	Incidence of chronic nephropathy,survival rate
**Dutra MF 2012**	Wistar rats	male	10	10	60%	17 week	120 day of age	Scr
**J Hempe 2012**	obese ZDF rats	male	8	5	70%	28 weeks	seven weeks of age	Scr,Urinary protein
**Keenan KP 2000**	SD rats	female	80	80	72%	104 week	7 week of age	Incidence of chronic nephropathy, Urinary protein
	SD rats	male	80	80	72%	104 week	7 week of age	Incidence of chronic nephropathy, Urinary protein
**Kobayashi S 1992**	F344 rats	male	6	6	60%	3 week	1 week after surgery	Urinary protein
**Kume S 2010**	C57BL/6 mice	male	10	10	60%	12 month	12 month of age	Urinary protein
**Lezcano EJ 2014**	ZDF rats	male	8	8	70%	8 week	week10-week19	Scr,Urinary protein
**Mark DA 1984**	MRL/1 MRL/n mice	male	10	9	50%	16week	1 month of age	BUN,Incidence of chronic nephropathy
**Masoro EJ 1989**	F344 rats	male	50	20	60%	6 months,12 months, 18 months,24 months	6 weeks of age	survival rate
**Nakano D 2011**	OLETF rats	male	16	16	70%	7 week	6 week of age	Urinary protein
**Nangaku M 2005**	SHR/NDmcr-cp (cp/cp) rats	male	5	5	70%	22 week	5 week of age	BUN, Scr, Urinary protein
**Yichun Ning 2013**	SD rats	male	8	8	60%	8 week	25 month of age	BUN, Scr
**Patricia R Johnson 1997**	Zucker rats	male	45	45	pair feed	Spontaneous death	4 week of age	Incidence of chronic nephropathy,survival rate
**Podkowka-Sieczka 2009**	Wistar rats	male	9	9	60%	12 month	7 week of age	Scr, Urinary protein
**Sell DR2000**	C57BL/6 mice	male	32	31	60%	Spontaneous death	508day of age	Incidence of chronic nephropathy,survival rate
**Shimokawa I 2003**	Wistar rats	male	29	30	70%	Spontaneous death	6 wk of age	Incidence of chronic nephropathy,survival rate
	transgenic wistar rats	male	30	30	70%	Spontaneous death	6 wk of age	Incidence of chronic nephropathy,survival rate
**TERESA A. DAVIS 1983**	Wistar rats	male	36	36	67%	2 years	32 days of age	Urinary protein,survival rate
	Wistar rats	male	36	36	67%	1 years	32 days of age	Urinary protein,survival rate
**Tikoo K 2007**	SD rats	male	6	6	fasting	8 week	1 week of acclimatization	BUN, Scr
**Wiggins JE 2005**	F344 rats	male	5	5	60%	5 to 7, 15 to 17, and 24 month	2 month of age	Urinary protein
**Zha Y 2008**	Wistar rats	male	30	30	70%	Spontaneous death	6 week of age	Incidence of chronic nephropathy,survival rate
**Sarah M. Tucker 1976**	Wistar rats	Male	12	11	60%~70%	8 month	4 month of age	Urinary protein
						20 month	4 month of age	Urinary protein
**Jennifer R. Wyndham 1983**	Wistar rats	male	6	6	60%	840 days	50 days of age	Urinary protein

SCR, serum creatinine; BUN, Blood urea nitrogen; GFR, glomerular filtration rate.

### 3.4 BUN (blood urea nitrogen)

**Fig 2:**
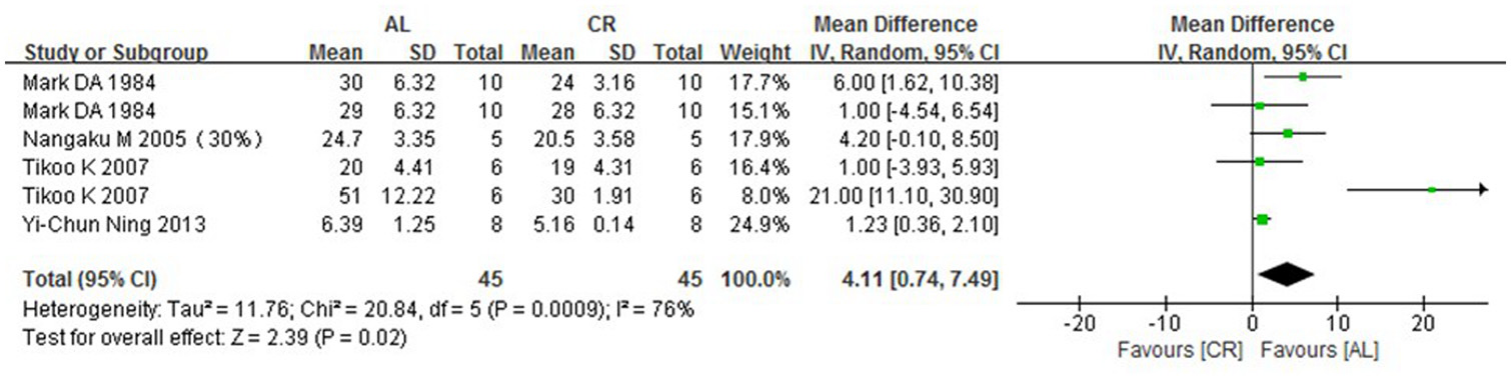
Forest plot comparing the blood urea nitrogen of the ad libitum—fed and caloric restriction groups. ■, WMD of each study; horizontal lines represent the 95% CIs for the data; ◆, combined overall effect.

Blood urea nitrogen was measured in six groups in 4 studies. Our analysis used the WMD method (weighted mean differences) along with a random effects model to show that the ad libitum—fed (AL) group showed a significantly higher BUN of 4.11 mg/dl (95% CIs: 0.74, 7.49; P = 0.02) compared with the caloric-restriction (CR) group (Fig 2).

### 3.5 Serum creatinine

**Fig 3:**
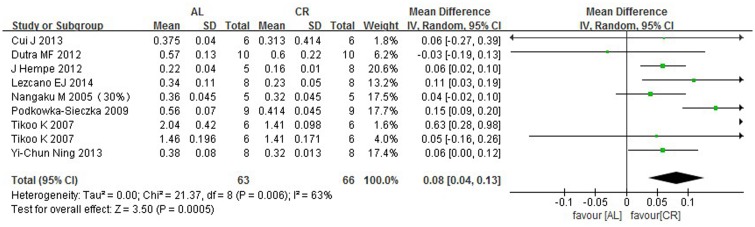
Forest plot comparing the serum creatinine levels of the ad libitum—fed and caloric restriction groups. ■, WMD of each study; horizontal lines represent the 95% CIs for the data; ◆, combined overall effect.

The serum creatinine levels were measured in nine groups in eight studies. The mean serum creatinine level was significantly lower, by 0.08 mg/dl (95% CIs: 0.04, 0.13; P = 0.0005), in the CR group compared with the AL group. The outcomes are shown in Fig 3.

### 3.6 Urinary protein

**Fig 4:**
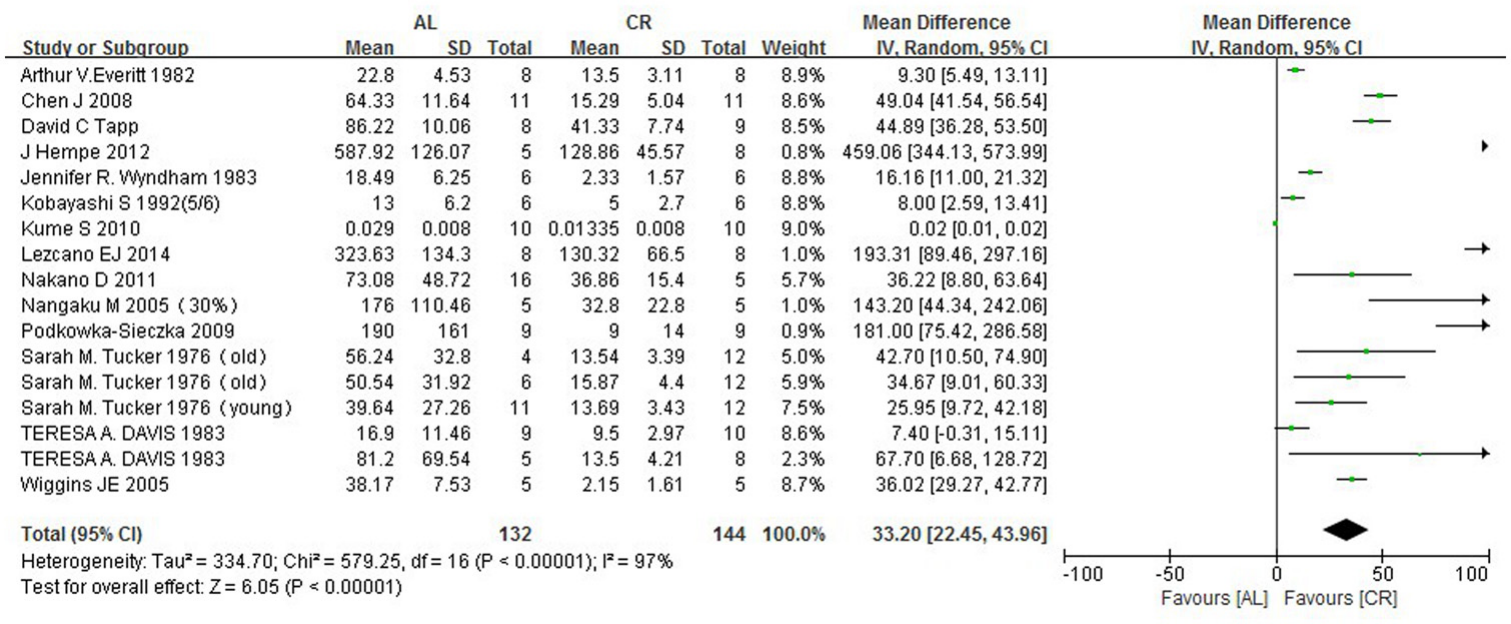
Forest plot comparing the urinary protein levels of the ad libitum—fed and caloric restriction groups. ■, WMD of each study; horizontal lines represent the 95% CIs for the data; ◆, combined overall effect.

When considering the 14 articles analyzing urinary protein and caloric restriction, significant statistical heterogeneity was observed because different models (age-related, CKD and diabetes) and different species were included in the studies (Fig 4). Therefore, we performed a random-effect analysis, but the statistical heterogeneity could not be eliminated (I^2^ = 97%). We also examined subgroups with different duration of caloric restriction, rodent strains, and animal models; nevertheless, the heterogeneity could not be eliminated. However, we showed that the urinary protein in the AL group was higher than that in the CR group by 33.20 mg/kg/24 h (95% CIs: 22.45, 43.96; P<0.00001).

### 3.7 Incidence of nephropathy

**Fig 5:**
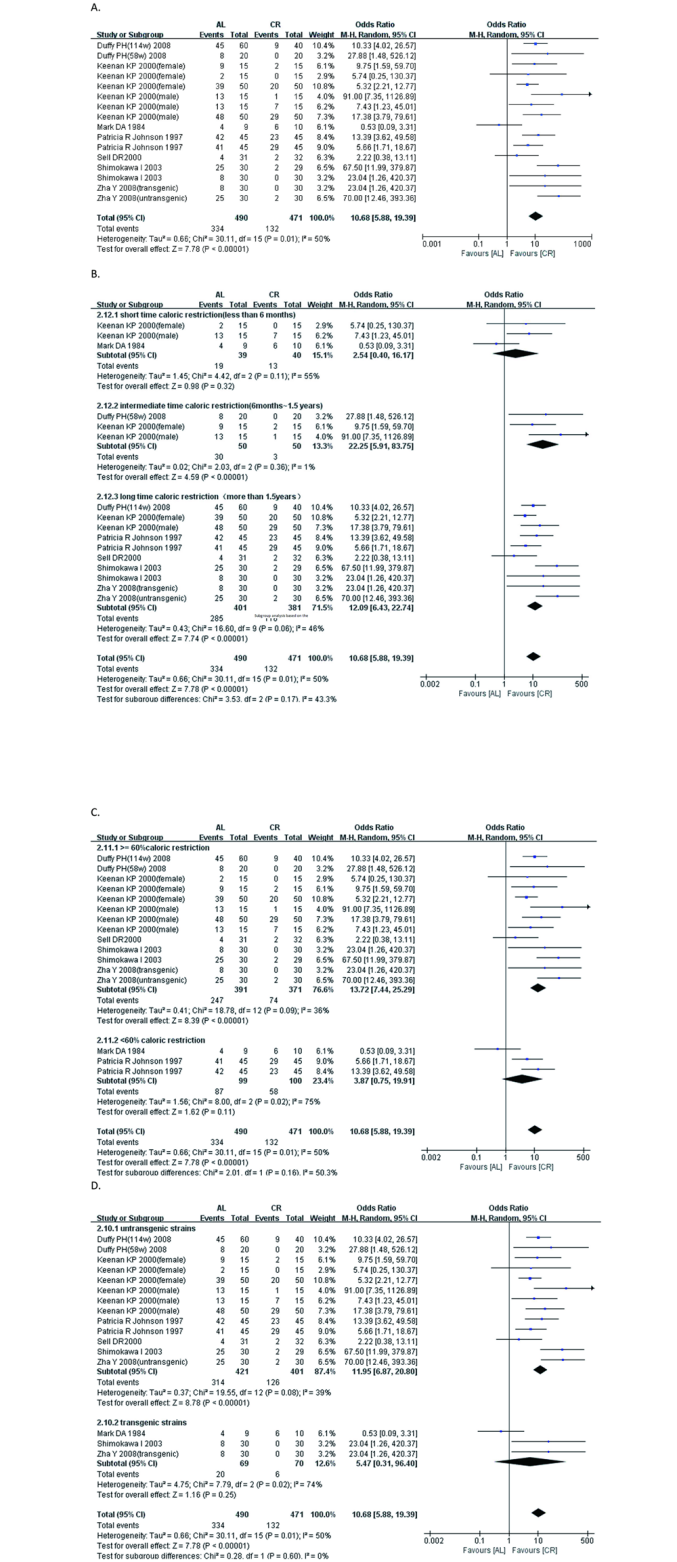
Forest plot comparing the incidence of nephropathy in the ad libitum—fed group and the caloric restriction group. ■, OR (odds ratios) of each study; horizontal lines represent the 95% CIs for the data; ◆, combined overall effect. (A) The effectiveness of the caloric restriction on the incidence of nephropathy. (B) Subgroup analysis based on the intervention duration. (C) Subgroup analysis based on the percentage of caloric restriction. (D) Subgroup analysis based on whether genetically modified model or not.

Nephropathy events are defined as the development and progression of glomerulosclerosis and tubulointerstitial glomerular lesions, which indicates that at least one morphological change, including tubular dilatation with protein casts, cysts, tubular epithelial degeneration and regeneration, chronic interstitial nephritis, and glomerular sclerosis, is taken into account. The incidence of nephropathy in the caloric restriction group was significantly lower than that in the ad libitum—fed (AL) group, as shown in Fig 5 (SMD: 10.68; 95% CIs: 5.88, 19.39; P<0.00001). In subgroups of different CR duration, we found that if the duration of caloric restriction was less than 6 months, no significant effect of intervention occurred. However, if the duration of caloric restriction was from six months to one and a half years, the incidence (OR) of kidney disease in the caloric restriction group was 22.25 (95% CIs, 5.91, 83.75; P<0.00001) lower than that in the AL group. If the duration of caloric restriction was greater than 1.5 years, the incidence of kidney disease in the caloric restriction group was 12.09 (95% CIs, 6.43, 22.74; P<0.00001) lower than that in the AL group. In accordance with the degree of caloric restriction, the beneficial effects of caloric restriction were more pronounced in groups in which the caloric intake level was less than 60% of that of the AL group. We then introduced genetically modified animals into the subgroup analysis. The OR for the wild-type group was 11.95 (95% CIs, 6.87, 20.80; P<0.00001), and the OR for the genetically modified group was 5.47 (95% CIs, 0.31, 96.40; P = 0.25). We used a funnel plot to evaluate reports for publication bias (S1 Fig). It exhibited symmetric patterns, we further use the Begg’s method and Egger’s method (comprehensive meta analysis 2.0 software) to confirm the results. The P-value of Egger’s regression intercept is 0.99(>0.05) and the P-value of Begg’s regression intercept is 0.93(>0.05) which indicated the absence of severe publication bias.

### 3.8 Survival rate (700~800 days)

**Fig 6:**
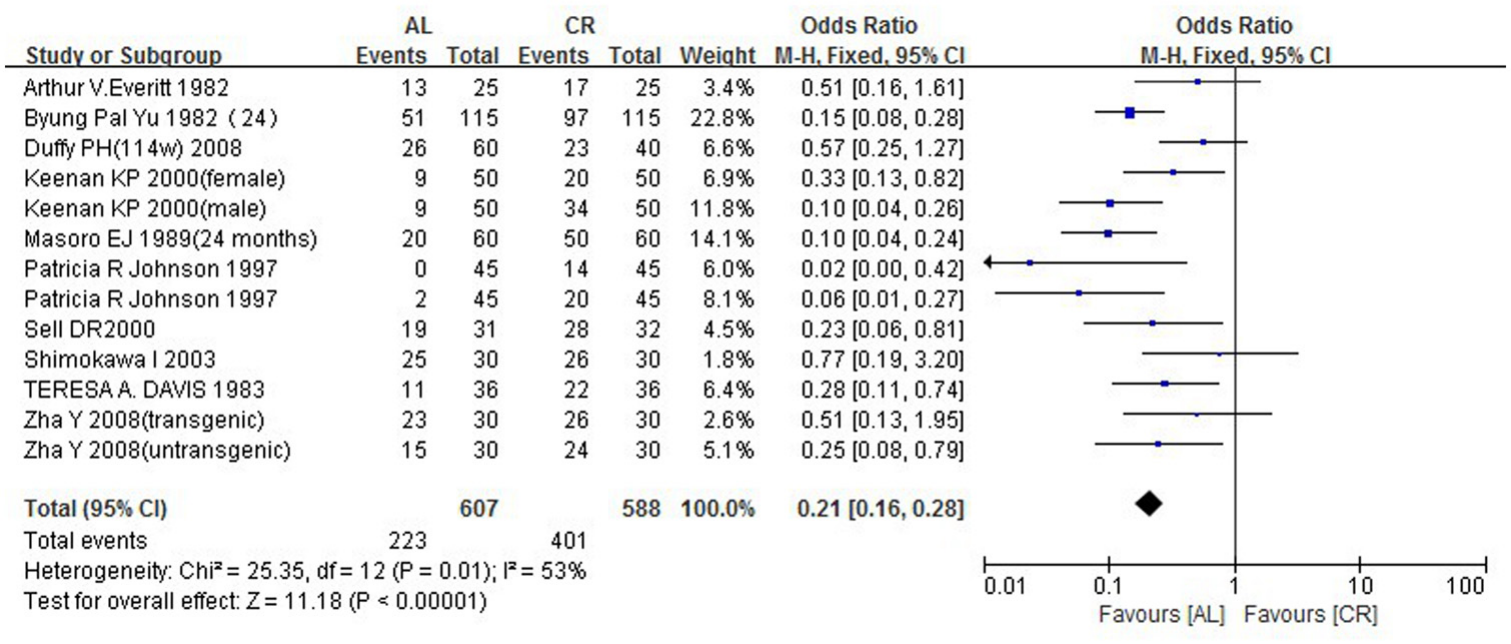
Forest plot comparing the survival rates of the ad libitum—fed and caloric restriction groups. ■, OR (odds ratios) of each study; horizontal lines represent the 95% CIs for the data; ◆, combined overall effect.

Ten articles showing that the survival rate of the caloric restriction group was much higher than that of the ad libitum—fed (AL) group (SMD: 0.21; 95% CIs: 0.16, 0.28; P<0.00001) were included in our meta-analysis (Fig 6).

## Discussion

A reliable conclusion of a meta-analysis is based on the quantity and quality of the included studies. Compared with clinical trials, the biases of systematic reviews are more serious for animal models. Based on the ARRIVE guidelines [15–17], the overall study quality was modest. Nineteen studies scored more than 4 points, which was regarded as “good methodological quality”, and the methodological quality scores ranged from 3 to 6. Strain difference is the first factor leading to bias in systematic reviews of animal experiments. Different species may have different susceptibilities to intervention and diverse basic metabolisms. These differences, however, may cause biases [18–19] and high heterogeneity. Because the studies meeting the inclusion criteria were so few, we could not fully divide the groups by strain. different eating habit may cause different metabolic balances of the in vivo, causing fluctuations in BUN. Additionally, eating habit also has the influence on the physical activities of the animals which can strongly disturbed Scr level. therefore, eating pattern may be the one causing the heterogeneity since restricting patterns of Tikoo study used the fasting method to restrict the caloric and other studies restricted the daily caloric intake instead of by fasting. In 24h urinary protein part, we use the meta regression to analysis the possible reason of high heterogeneity (S3 File). We exclude the different model(diabetes, aging disease), different species (Wistar Rat, F344 Rat, other species), publish date, intervention duration, the percentage of caloric restriction which the p value of meta regression are all over 0.05. Considering the time span and regional span of the included studies, we speculate that the different test method, changes of artificial feeding conditions and the varying food component may be the possible reason leading to potential heterogeneity. Besides, since our statistical data is the 24h urinary protein, the method of collecting sample play the most significant role in the high heterogeneity. Different duration or procedure of urine collection in the included studies may cause the urinary concentration or incompleted urinary sample resulting in the heterogeneity. Therefore, a random effects analysis was performed, and subgroups were made to decrease heterogeneity, leading to more consistent results.

Increases in urea nitrogen, creatinine and urinary protein are the most commonly used markers of kidney function. The levels of BUN and Scr increase with the occurrence and progression of diabetic nephropathy [20]. In addition, high 24 h urine protein has been considered as a high risk factor affecting kidney function in patients with CKD. In more than 50 years of clinical practice, low protein diets have been proposed to patients with kidney failure. However, the effects of these diets in preventing severe kidney failure are unclear and the need for maintenance dialysis has not been resolved. Low protein diets have been proposed to patients with kidney failure for a long time. However, the effects of these diets in preventing severe kidney failure have not been resolved. Reducing protein intake in patients with CKD reduces the occurrence of renal death by 32% compared with higher or unrestricted protein intake. The optimal level of protein intake cannot be confirmed from these studies [21]. In a long-term follow-up study [22], it was found that a very low-protein diet did not delay the progression to kidney failure, but it appeared to increase the risk of death. Whereas the value of a very low-protein diet in CKD patients is questionable, caloric restriction provides a new possibility. Through the meta-analysis of cross-sectional and prospective longitudinal studies, we found a significant preservation of kidney function in the caloric restriction group, as indicated by markers including BUN, Scr Compared with the AL group, the urea nitrogen, creatinine and urinary protein levels of the CR group were significantly lower, by 4.11 mg/dl (95% CIs: 0.74, 7.49; P = 0.02), 0.08 mg/dl (95% CIs: 0.04, 0.13; P = 0.0006) and 33.20 mg/kg/24 h (95% CIs: 22.45, 43.96; P<0.00001), respectively.

The occurrence of morphological changes is an end-stage event in CKD. We defined nephropathy events as the development and progression of glomerulosclerosis and tubulointerstitial glomerular lesions, which indicates that at least one morphological change, including tubular dilatation with protein casts, cysts, tubular epithelial degeneration and regeneration, chronic interstitial nephritis, and glomerular sclerosis. Pooled analyses of all of the studies revealed that the incidence of CKD in the AL group was 10.68 times higher than that in the CR group. Therefore, the hypothesis that CR can delay the occurrence of CKD was strongly supported by our analysis.

How much restriction is needed? How long should restriction continue? Answers to these questions are needed. If the intervention time and duration is inappropriate, researchers may construct an unsuccessful model, wasting time and money. Additionally, understanding the appropriate extent and duration of restriction may provide a good platform with which to evaluate the effects of CR in patients with CKD. Here, we conducted subgroup analyses based on the length of caloric restriction, the extent of food restriction extent and the type of animal model. Through our analysis, we observed that the effect of caloric restriction on the occurrence of kidney disease was only significant with prolonged intervention (more than 6 months), indicating that to maximize the profit potential of CR, the restricted duration should be as long as possible. In addition, the beneficial effects of CR on the 60%-caloric-restriction group (i.e., receiving 40% fewer calories than the AL group) were greater than on the less-than-60%-caloric-restriction group, which showed no significant differences from the AL group in nephropathy events. One possible reason for this difference between the two caloric-restriction groups is that low caloric intake may cause malnutrition and accelerate the occurrence of renal events. Previous studies have also shown that 80% caloric restriction (20% less than the AL group) or more may not show obvious effects of CR. In many CR protocols with rodents, the control group is restricted by 10% relative to true ad libitum intake. Therefore, to obtain optimal benefits, 60~80% caloric intake is more effective. Finally, caloric restriction did not show obvious protective effects in genetically modified strains, possibly because genetic changes may lead to invalid caloric restriction molecular pathways, which further influence the effect of caloric restriction. However, our analysis of subgroups suggest that mild, extended restriction can maximize the effect of CR, which may help researchers construct successful and optimal rodent models of CR. These results may also inform the preparation of a communal CR therapeutic regimen in the future.

Survival rate is also a powerful endpoint of caloric restriction. Previous studies have revealed the role of caloric intake on longevity [18,23]; therefore, we analyzed survival rate in this meta-analysis. We selected survival rates of 700~800 days and found that, within this time frame, the survival rate of the caloric restriction group was much higher than that of the ad libitum—fed (AL) group (SMD: 0.21; 95% CIs: 0.16, 0.28; P<0.00001), consistent with previous studies [11,16,24].

While the impact of caloric restriction on human health is not fully understood, there is strong evidence to support further studies of its influence on the treatment of chronic diseases. In recent years, researchers have taken solid steps towards this target. To date, detailed clinical research on the effects of CR against CKD in humans is lacking. Morales [25] studied the relationship between weight loss and chronic protein uric nephropathies. They randomly assigned 30 overweight and obese adults with proteinuria from diabetic and non-diabetic kidney diseases into groups consuming either a normal diet or a CR diet. After 5 months of caloric restriction, proteinuria decreased by over 30% from a mean of 2.8 g/day at baseline to 1.9 g/day. Similar results were obtained by Giordani and his colleague [26]. They evaluated 14 patients with type 2 diabetes mellitus, morbid obesity and stage 2 CKD before and after a 7-day, very-low-calorie diet (VLCD). After the VLCD, Renal function, as measured by glomerular filtration rate, increased significantly. These experiments are, to some extent, in accord with the results of our analysis: caloric restriction benefitted renal function. Although our meta-analysis and related clinical research [27–31] have shown promising and powerful effects of CR against CKD, strong conclusions cannot be drawn because of the more complicated internal environment of humans relative to rodent species. Much work remains to fully understand the effects of CR diet strategies.

## Conclusions

Based on the results of this meta-analysis, we infer that caloric restriction can delay the progression of CKD by decreasing the levels of urea nitrogen, creatinine, and urine protein and enhance the survival rate of rodent models by reducing disease onset especially CKD. Moreover, the extent of restriction and the time of intervention may play important roles in suppressing the development and progression of CKD. Presently, much more will need to be done before CR will be formally applied to treat disease. The extremely complex internal environment of humans, patient compliance issues and diverse eating habits challenge the progress of the research. Nevertheless, through conducting this review, we believe CR may become a promising method to cure CKD, and we are hopeful that CR can soon be used to benefit humans.

## Supporting Information

S1 Fig Funnel plot of 16 trials assessing the effect of CR against the occurrence of CKD. Funnel plot of 16 trials exhibits symmetric patterns demonstrating that there is no evidence of publication bias in the included studies.
(TIF)

S1 File PRISMA checklist. (DOC)

S2 File Included studies in the meta analysis. 27 citations are included in the meta-analysis.
(DOCX)

S3 File The data of analyzing the possible reason causing the high heterogeneity in 24h urinary protein. we use the meta regression to analysis the possible reason of high heterogeneity. We exclude the different model (diabetes, aging disease), different species (Wistar Rat, F344 Rat, other species), publish date, intervention duration, the percentage of caloric restriction.
(DOCX)
