# Supplementary figures and images for: Beneficial Effects of Caloric Restriction on Chronic Kidney Disease in Rodent Models: A Meta-Analysis and Systematic Review
Source: PLoS One. 2015 Dec 22;10(12):e0144442. doi: 10.1371/journal.pone.0144442 (PMC4690609; doi:10.1371/journal.pone.0144442)

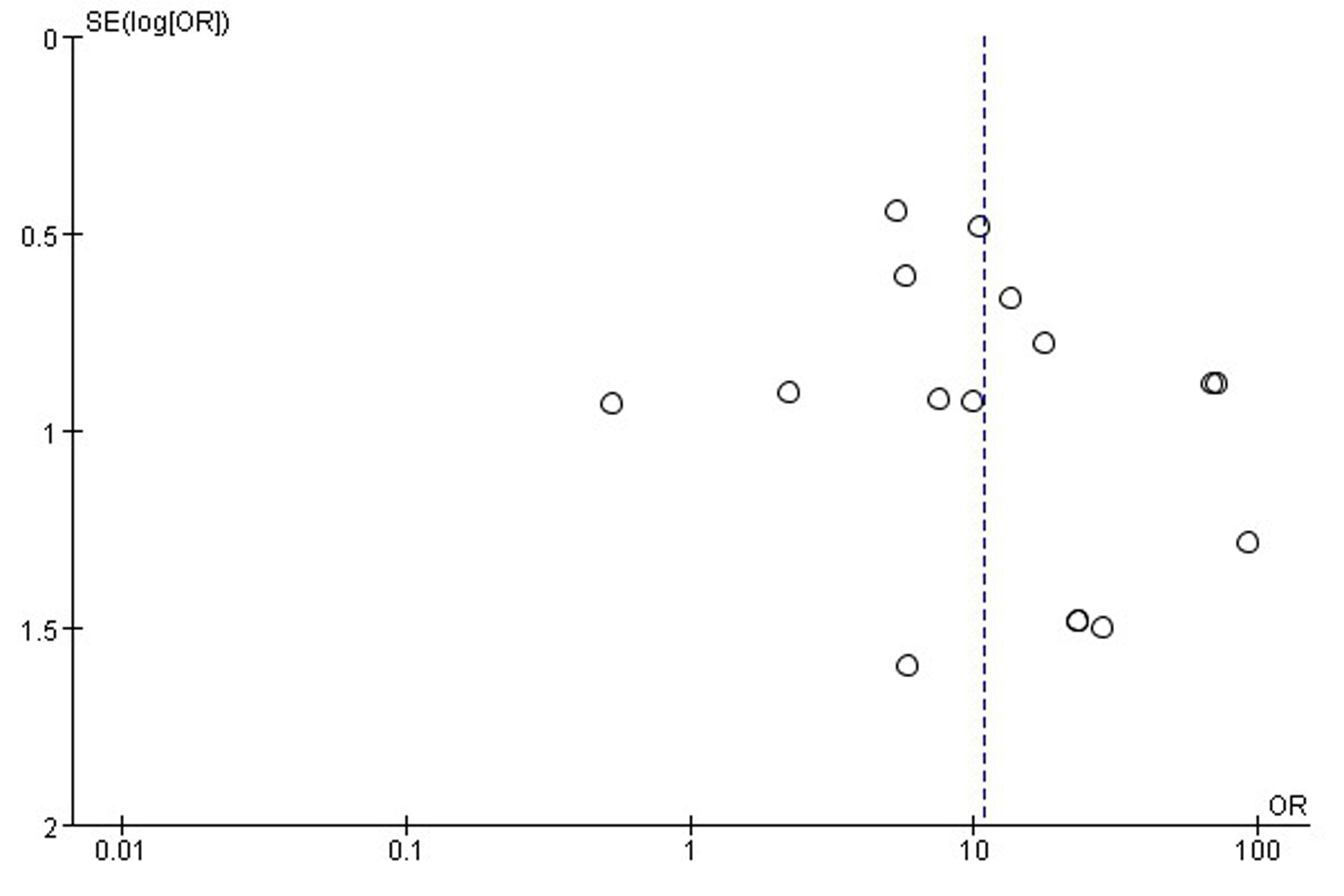

Supplement: S1 Fig — Funnel plot of 16 trials exhibits symmetric patterns demonstrating that there is no evidence of publication bias in the included studies. (TIF) [file pone.0144442.s001.tif]
