# Supplementary material for: Beneficial Effects of Caloric Restriction on Chronic Kidney Disease in Rodent Models: A Meta-Analysis and Systematic Review
Source: PLoS One. 2015 Dec 22;10(12):e0144442. doi: 10.1371/journal.pone.0144442 (PMC4690609; doi:10.1371/journal.pone.0144442)
Supplement: S2 File — 27 citations are included in the meta-analysis. (DOCX) [file pone.0144442.s003.docx]

**Included studies**

1. Velalar CN, Chen J, Ruan R. Identifying the changes in gene profiles regulating the amelioration of age-related oxidative damages in kidney tissue of rats by the intervention of adult-onset calorie restriction. Rejuvenation Res. 2008;11(4):757-63.

2. Cui J, Shi S, Sun X, Cai G, Cui S, Hong Q, et al. Mitochondrial autophagy involving renal injury and aging is modulated by caloric intake in aged rat kidneys. PloS one. 2013;8(7):e69720.

3. Davis TA, Bales CW, Beauchene RE. Differential effects of dietary caloric and protein restriction in the aging rat. Exp Gerontol. 1983;18(6):427–35.

4. PH D, SM L, MA M, RW T, BS H, BT T, et al. Nonneoplastic pathology in male Sprague-Dawley rats fed the American Institute of Nutrition-93M purified diet at ad libitum and dietary-restricted intakes. Nutr Res. 2008;28(3):179–89.

5. MF D, IJ B, C B, NB C, AF V, DF dS, et al. Effects of chronic caloric restriction on kidney and heart redox status and antioxidant enzyme activities in Wistar rats. BMB reports. 2012;45(11):671-6.

6. AV E, BD P, JR. W. Effects of caloric intake and dietary composition on the development of proteinuria, age-associated renal disease and longevity in the male rat. Gerontology. 1982;28(3):168-75.

7. J H, R E, HL S, W K, AW H. Appropriateness of the Zucker Diabetic Fatty rat as a model for diabetic microvascular late complications. Laboratory Animals. 2012;46(1):32-9.

8. PR J, JS S, BA H, Jr HR, SF G. Longevity in obese and lean male and female rats of the Zucker strain: prevention of hyperphagia. Am J Clin Nutr. 1997;66(4):890-903.

9. KP K, JB C, CL M, CM H, KA S, P. L. Chronic nephropathy in ad libitum overfed Sprague-Dawley rats and its early attenuation by increasing degrees of dietary (caloric) restriction to control growth. Toxicologic Pathology. 2000;28(6):788-98.

10. S K, MA. V. Differential effects of calorie restriction on glomeruli and tubules of the remnant kidney. Kidney Int. 1992;42(3):710–7.

11. Kume S, Uzu T, Horiike K, Chin-Kanasaki M, Isshiki K, Araki S-i, et al. Calorie restriction enhances cell adaptation to hypoxia through Sirt1-dependent mitochondrial autophagy in mouse aged kidney. The Journal of Clinical Investigation. 2010;120(4):1043-55.

12. Lezcano EJ, Iñigo P, Larraga AM, Barranquero C, Gimenez I, Osada J. Caloric restriction or telmisartan control dyslipidemia and nephropathy in obese diabetic Zücker rats. Diabetology &amp; Metabolic Syndrome. 2014;6(2):217-24.

13. Mark DA, Alonso DR, Quimby F, Thaler HT, Kim YT, Fernandes G, et al. Effects of nutrition on disease and life span. I. Immune responses, cardiovascular pathology, and life span in MRL mice. The American journal of pathology. 1984;117(1):110-24.

14. EJ M, K I, CA G, CA M, EJ S, BP. Y. Dietary modulation of the progression of nephropathy in aging rats: an evaluation of the importance of protein. The American Journal of Clinical Nutrition. 1989;49(6):1217-27.

15. D N, S D, K K, H H, H M, T M, et al. Short-Term Calorie Restriction in Early Life Attenuates the Development of Proteinuria but Not Glucose Intolerance in Type 2 Diabetic OLETF Rats. ISRN Endocrinology. 2011;2011.

16. Nangaku M, Izuhara Y, Usuda N, Inagi R, Shibata T, Sugiyama S, et al. In a type 2 diabetic nephropathy rat model, the improvement of obesity by a low calorie diet reduces oxidative/carbonyl stress and prevents diabetic nephropathy. Nephrology, dialysis, transplantation : official publication of the European Dialysis and Transplant Association - European Renal Association. 2005;20(12):2661-9.

17. Ning YC, Cai GY, Zhuo L, Gao JJ, Dong D, Cui S, et al. Short-term calorie restriction protects against renal senescence of aged rats by increasing autophagic activity and reducing oxidative damage. Mechanisms of ageing and development. 2013;134(11-12):570-9.

18. R P-S, K W-T, ZI N, A S, A B, DG. O. The effect on renal structure and function of late-life-introduced caloric restriction (CR) in rats. International Urology and Nephrology. 2009;41(1):211-7.

19. DR S, NR K, VM. M. Longitudinal determination of skin collagen glycation and glycoxidation rates predicts early death in C57BL/6NNIA mice. FASEB journal : official publication of the Federation of American Societies for Experimental Biology. 2000;14(1):145-56.

20. I S, Y H, T T, H O, T K, T C, et al. Life span extension by reduction of the growth hormone-insulin-like growth factor-1 axis: relation to caloric restriction. The FASEB Journal. 2003;17(6):1108-9.

21. DC T, WG W, JF A, DN H, JL B, MA V. Food restriction retards body growth and prevents end-stage renal pathology in remnant kidneys of rats regardless of protein intake. Laboratory investigation; a journal of technical methods and pathology. 1989;60(2):184-95.

22. Tikoo K, Tripathi DN, Kabra DG, Sharma V, Gaikwad AB. Intermittent fasting prevents the progression of type I diabetic nephropathy in rats and changes the expression of Sir2 and p53. FEBS letters. 2007;581(5):1071-8.

23. SM T, RL M, RE B. Influence of diet and feed restriction on kidney function of aging male rats. Journal of Gerontology. 1976;31(3):264-70.

24. JE W, M G, SK S, BL W, KA S, DE M, et al. Podocyte hypertrophy, "adaptation," and "decompensation" associated with glomerular enlargement and glomerulosclerosis in the aging rat: prevention by calorie restriction. Journal of the American Society of Nephrology. 2005;16(10):2953-66.

25. Wyndham J, Everitt A, Everitt S. Effects of isolation and food restriction begun at 50 days on the development of age-associated renal disease in the male Wistar rat. Arch Gerontol Geriatr. 1983;2.

26. BP Y, EJ M, I M, HA B, FT L. Life span study of SPF Fischer 344 male rats fed ad libitum or restricted diets: longevity, growth, lean body mass and disease. Journal of gerontology. 1982;37(2):130-41.

27. Y Z, T T, A N, I S, Y H, MS R. Genetic Suppression of GH-IGF-1 Activity, Combined with Lifelong Caloric Restriction, Prevents Age-Related Renal Damage and Prolongs the Life Span in Rats. Am J Nephrol 2008. 2008;28(5):755-64.
