# Supplementary material for: Beneficial Effects of Caloric Restriction on Chronic Kidney Disease in Rodent Models: A Meta-Analysis and Systematic Review
Source: PLoS One. 2015 Dec 22;10(12):e0144442. doi: 10.1371/journal.pone.0144442 (PMC4690609; doi:10.1371/journal.pone.0144442)

A.meta regression based on different models


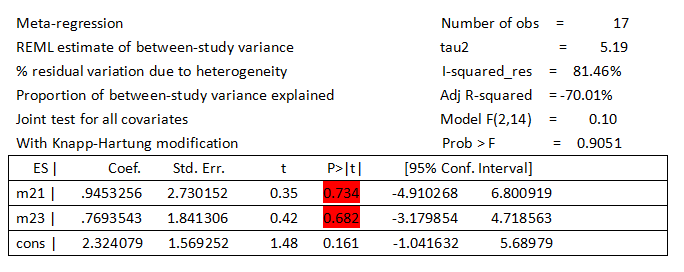


B.meta regression based on different species


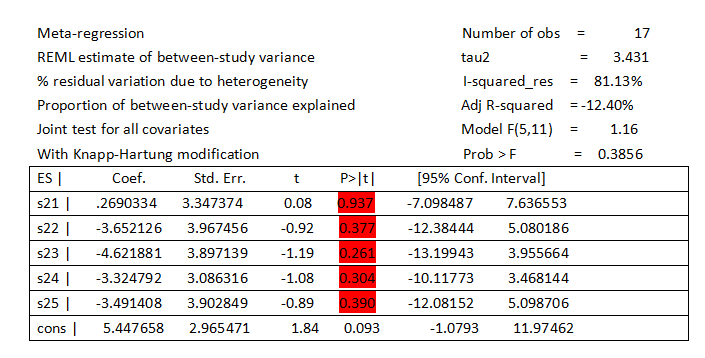


C.meta regression based on publish date


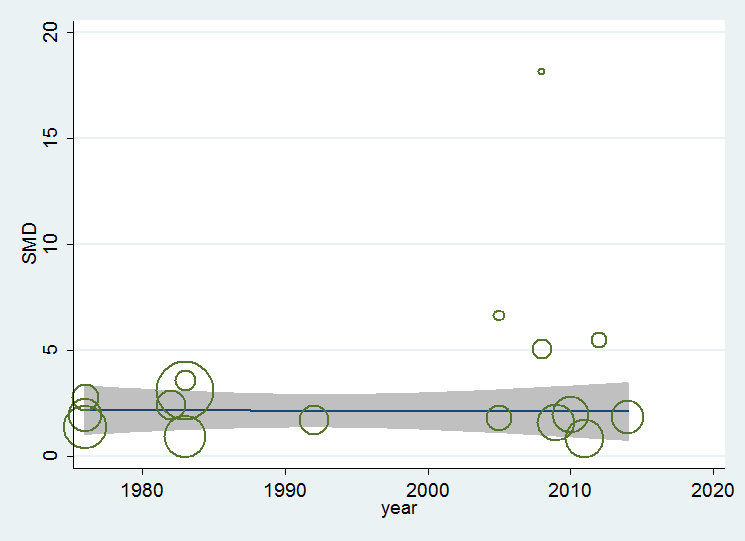


D.meta regression based on intervention duration


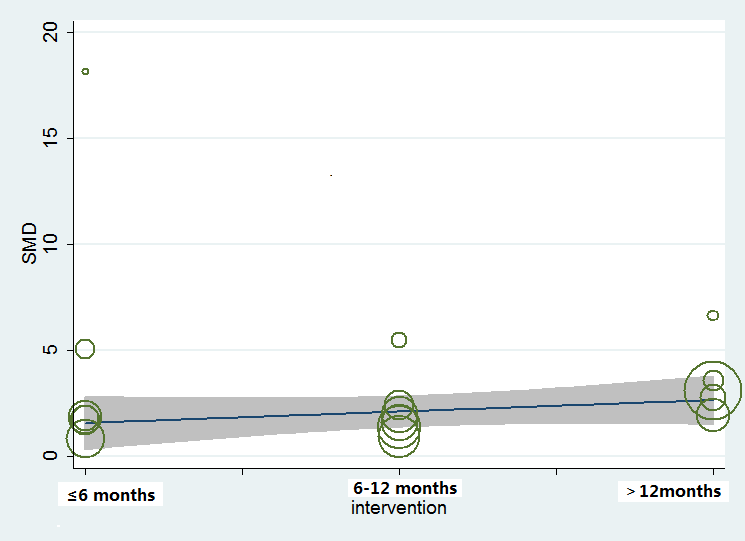


E. meta regression based on the percentage of caloric restriction


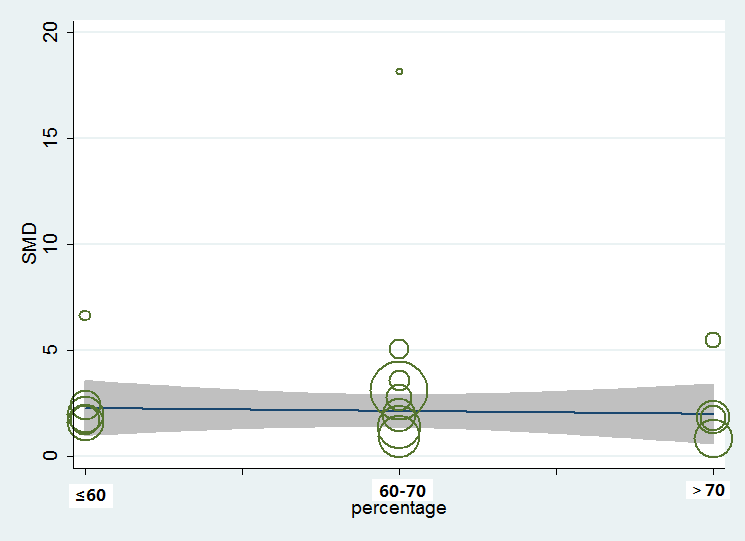

Supplement: S3 File — we use the meta regression to analysis the possible reason of high heterogeneity. We exclude the different model (diabetes, aging disease), different species (Wistar Rat, F344 Rat, other species), publish date, intervention duration, the percentage of caloric restriction. (DOCX) [file pone.0144442.s004.docx]
